# Predictors of survival in patients with influenza pneumonia-related severe acute respiratory distress syndrome treated with prone positioning

**DOI:** 10.1186/s13613-018-0440-4

**Published:** 2018-09-24

**Authors:** Kuo-Chin Kao, Ko-Wei Chang, Ming-Cheng Chan, Shinn-Jye Liang, Ying-Chun Chien, Han-Chung Hu, Li-Chung Chiu, Wei-Chih Chen, Wen-Feng Fang, Yu-Mu Chen, Chau-Chyun Sheu, Ming-Ju Tsai, Wann-Cherng Perng, Chung-Kan Peng, Chieh-Liang Wu, Hao-Chien Wang, Kuang-Yao Yang, TSIRC (Taiwan Severe Influenza Research Consortium)

**Affiliations:** 1 Department of Thoracic Medicine, Chang Gung Memorial Hospital, Taoyuan, Taiwan; 2 Department of Respiratory Therapy, Chang Gung University College of Medicine, Taoyuan, Taiwan; 3 Division of Chest Medicine, Department of Internal Medicine, and Section of Critical Care and Respiratory Therapy, Taichung Veterans General Hospital, Taichung, Taiwan; 4 Central Taiwan University of Science and Technology, Taichung, Taiwan; 5 Division of Pulmonary and Critical Care, Department of Internal Medicine, China Medical University Hospital, Taichung, Taiwan; 6 Division of Chest Medicine, Department of Internal Medicine, National Taiwan University Hospital, Taipei, Taiwan; 7 Department of Chest Medicine, Taipei Veterans General Hospital, No. 201, Section 2 Shi-Pai Road, Taipei, 11217 Taiwan; 8 Institute of Emergency and Critical Care Medicine, School of Medicine, National Yang-Ming University, Taipei, Taiwan; 9 Division of Pulmonary and Critical Care Medicine, Department of Internal Medicine, Kaohsiung Chang Gung Memorial Hospital, Kaohsiung, Taiwan; 10 Department of Respiratory Care, Chang Gung University of Science and Technology, Chiayi, Taiwan; 11 Division of Pulmonary and Critical Care Medicine, Kaohsiung Medical University Hospital, Kaohsiung, Taiwan; 12 School of Medicine, College of Medicine, Kaohsiung Medical University, Kaohsiung, Taiwan; 13 Division of Pulmonary and Critical Care Medicine, Department of Internal Medicine, Tri-Service General Hospital, National Defense Medical Center, Taipei, Taiwan; 14 Center for Quality Management, Taichung Veterans General Hospital, Taichung, Taiwan; 15 Office of Medical Administration, Taichung Veterans General Hospital, Taichung, Taiwan

**Keywords:** ARDS, Prone positioning, Influenza, Driving pressure, Mortality

## Abstract

**Background:**

Patients with influenza complicated with pneumonia are at high risk of rapid progression to acute respiratory distress syndrome (ARDS). Prone positioning with longer duration and lung-protective strategies might reduce the mortality level in ARDS. The aim of this study is to investigate the survival predictors of prone positioning in patients with ARDS caused by influenza pneumonia.

**Methods:**

This retrospective study was conducted by eight tertiary referral centers in Taiwan. From January 1 to March 31 in 2016, all of the patients in intensive care units with virology-proven influenza pneumonia were collected, while all of those patients with ARDS and receiving prone positioning were enrolled. Demographic data, laboratory examinations, management records, ventilator settings and clinical outcomes were collected for analysis.

**Results:**

During the study period, 336 patients with severe influenza pneumonia were screened and 263 patients met the diagnosis of ARDS. Totally, 65 patients receiving prone positioning were included for analysis. The 60-day survivors had lower Acute Physiology and Chronic Health Evaluation (APACHE) II score, pneumonia severity index (PSI), creatinine level and lower rate of receiving renal replacement therapy than non-survivors (22.4 ± 8.5 vs. 29.2 ± 7.4, *p* = 0.003; 106.6 ± 40.9 vs. 135.3 ± 48.6, *p* = 0.019; 1.2 ± 0.9 mg/dL vs. 3.1 ± 3.6 mg/dL, *p* = 0.040; and 4% vs. 42%, *p* < 0.005). Multivariate Cox regression analysis identified PSI (hazard ratio 1.020, 95% confidence interval 1.009–1.032; *p* < 0.001), renal replacement therapy (hazard ratio 6.248, 95% confidence interval 2.245–17.389; *p* < 0.001), and increase in dynamic driving pressure (hazard ratio 1.372, 95% confidence interval 1.095–1.718; *p* = 0.006) which were independent predictors associated with 60-day mortality.

**Conclusions:**

In the present study, in evaluating the effect of prone positioning in patients with influenza pneumonia-related ARDS, pneumonia severity index, renal replacement therapy and increase in dynamic driving pressure were associated with 60-day mortality in patients with influenza pneumonia-related ARDS receiving prone positioning.

**Electronic supplementary material:**

The online version of this article (10.1186/s13613-018-0440-4) contains supplementary material, which is available to authorized users.

## Background

Severe complicated influenza including pneumonia, myocarditis and neurologic complications are still a burden on intensive care units (ICU) nowadays, especially viral or secondary bacteria pneumonia-induced acute respiratory distress syndrome (ARDS) [1, 2]. During the winter season in 2016, there was an outbreak of influenza in Taiwan. Totally, 1735 subjects were admitted to ICUs due to severe complicated influenza pneumonia according to the data from the Centers for Disease Control of Taiwan [3]. Patients with influenza pneumonia needing mechanical ventilation were at high risk of rapid progression to ARDS. For the 2009 pandemic H1N1 virus infection, 49–72% of patients admitted to ICUs had complications with ARDS [4, 5].

There are several therapeutic options for refractory hypoxemia in patients with severe ARDS [6, 7], but only a few options have been confirmed with clinical validity by previous studies, including higher positive end-expiratory pressure (PEEP) [8, 9], lower tidal volume [10], neuromuscular blocking agents [11] and prone positioning [12]. Prone positioning was first suggested in 1974 [13]; however, the clinical benefit of prone positioning in patients with ARDS was not confirmed until 2013 when the PROSEVA study showed decreased 28-day and 90-day mortality and increased ventilator-free days only when it was started early and there were sufficiently long sessions [12]. Further, meta-analysis by Cochrane database also revealed that prone positioning would reduce the mortality rate when used with lung-protective strategies and longer duration in patients with severe ARDS [14, 15].

Few studies have explored the effect of prone positioning focused on influenza pneumonia-related ARDS patients. Xu et al. [16] studied H7N9 influenza patients with prone positioning, and decrease in carbon dioxide retention was noted, but no clinical outcome was mentioned. Moreover, what factors that can predict the efficacy of prone positioning in severe ARDS are not entirely clear [17].

The aim of this study is to investigate the survival predictors of prone positioning in patients with severe ARDS caused by influenza pneumonia.

## Methods

### Study population and data collection

This multicenter retrospective cohort study was conducted by the Taiwan Severe Influenza Research Consortium (TSIRC), which included eight tertiary referral centers (four hospitals in northern Taiwan, two hospitals in central Taiwan and two hospitals in southern Taiwan). Over a period of 3 months from January 1 to March 31 in 2016, all patients with the virology-proven influenza infection who were admitted to ICUs due to severe complicated influenza in these eight hospitals were collected and their data were analyzed. All patients diagnosed as severe ARDS according to Berlin definition and also receiving prone positioning were collected for investigation [18]. The Berlin definition of ARDS was defined by acute onset within 1 week, bilateral lungs opacities, no evidence of cardiac failure-related hydrostatic edema by echocardiography, and PaO_2_/FiO_2_ ratio < 300 mm Hg with positive end-expiratory pressure (PEEP) ≥ 5 cm H_2_O. The demographic and laboratory data, treatment record, mechanical ventilation settings, and clinical outcomes were analyzed from the electronic medical records with a standardized case report form in each hospital. The Ethical Committee/Institutional Review Board for Human Research of the involved hospitals approved this study (Chang Gung Memorial Hospital 201600988B0, Taichung Veterans General Hospital CE16093A, Taipei Veterans Hospital CE16093A, Taipei Veterans General Hospital 2016-05-020CC, Kaohsiung Medical University Hospital KUMHIRB-E(I)-20170097, Kaohsiung Chang-Gung Memorial Hospital 201600988B0, China Medical University Hospital 105-REC2-053 (FR), National Taiwan University Hospital 201605036RIND, National Taiwan University Hospital 201605036RIND, Tri-Service General Hospital 1-105-05-086). The need for informed consent was waived, and patients’ data were anonymized and de-identified prior to analysis.

### Confirmation of influenza infection

Influenza infection was confirmed by one of the following tests revealing as positive including the rapid antigen test, nucleic acid reverse transcriptase polymerase chain reaction (RT-PCR), viral culture sampling from nasopharynx swab, throat swab, sputum or bronchoalveolar lavage and positive serum antibody serologic test (antibody titers increased more than 4 times from acute to convalescent stages).

### Mechanical ventilator settings

The usual practice in the units was that patients be ventilated with lung-protective strategy by low tidal volume 6–8 mL/kg of predict body weight plus low positive end-expiratory pressure (PEEP)–oxygen fraction in air (FiO_2_) table for pressure-controlled or volume-controlled ventilation [10]. Ventilation was monitored by arterial blood gas measurements, with ventilator settings changed as needed. Pulse oximetry (SpO_2_) was used to monitor oxygenation, and ventilatory settings were adjusted to maintain SpO_2_ > 90% or PaO_2_ > 60 mm Hg and to avoid raising the plateau pressure > 30 cm H_2_O.

### Prone positioning

The method of prone positioning complied with the PROSEVA study [12]. Doses of neuromuscular blocking agent with intravenous cisatracurium and sedatives with intravenous midazolam were adjusted to maintain synchrony between the ventilator and the patient’s breathing, as well as hemodynamics. The criteria for stopping prone positioning were any of the following: improvement in oxygenation (defined as a PaO_2_/FiO_2_ ratio ≥ 150 mm Hg, with a PEEP of ≤ 10 cm H_2_O and an FiO_2_ ≤ 0.6), a decrease in the PaO_2_/FiO_2_ ratio ≥ 20% or complications happening during prone positioning such as SpO_2_ ≤ 85% or PaO_2_/FiO_2_ ratio ≤ 55 mm Hg, severe cardiac arrhythmia, systolic blood pressure ≤ 60 mm Hg and any other life-threatening condition for which the intensivist decided to stop the prone positioning.

### Laboratory data

The laboratory data including baseline characteristics, underlying disease, complete blood count, differential count and biochemistry data were obtained when the patient was admitted to the ICU. The mechanical ventilator settings were recorded such as peak inspiratory pressure, PEEP, artery blood gas, partial pressure of oxygen in arterial blood (PaO_2_), PaO_2_/FiO_2_ ratio, tidal volume, dynamic driving pressure and dynamic compliance of the respiratory system before and 1 day after the first prone positioning. The above physiological data were recorded before prone positioning on the supine position and 1 day after first prone positioning on the prone position. The dynamic driving pressure and dynamic compliance were computed as peak pressure minus PEEP and tidal volume divided by peak pressure minus PEEP. The severity scores including pneumonia severity index (PSI) [19], Acute Physiology and Chronic Health Evaluation II (APACHE II) score [20], CURB-65 (Confusion, Urea > 7 mmol/L, Respiratory rate ≥ 30/min, Blood pressure [systolic < 90 mm Hg or diastolic ≤ 60 mm Hg] and age ≥ 65 years) pneumonia severity score [21] and Sequential Organ Failure Assessment (SOFA) score [22] were collected on the ICU admission day.

### Statistical analyses

Statistical analyses and database management were performed using SPSS version 17.0.0 (SPSS Inc., Chicago, IL). The data were presented as number (percentages) for nominal variables, and as mean ± standard deviation for continuous variables. The chi square test was used to compare the nominal variables, and the Student’s *t* test was used to compare the continuous variables. Cox proportional hazard models were used with covariates significantly different between survivors and non-survivors at the threshold of 0.2 and mortality at day 60 as the dependent variable. Calibration was assessed using Hosmer–Lemeshow goodness-of-fit test (C statistic, goodness of fit was defined as a *p* value > 0.05), and discrimination was assessed by the area under the receiver operating curves. Even though peak airway pressure, dynamic driving pressure, and compliance are mathematically coupled, we planned to formally test the collinearity within them and, if verified, to use a specific Cox model for each. We also included those collinear variables two-by-two into three additional Cox regression models [23], besides the other covariates. One model pertained to peak airway pressure and dynamic driving pressure, one to peak airway pressure and compliance, and one to dynamic driving pressure and compliance. If both variables in the couple lacked significance, the conclusion could be that the same information was carried by each component of the couple. If one of the variables in the couple remained significantly correlated with survival, this variable would be more informative than the other in the couple. Univariate and multivariate Cox proportional hazard regression models were used to estimate the hazard ratio (HR). In this study, we used the two-tailed test, and the definition of significance was *p* value < 0.05.

## Results

**Fig. 1:**
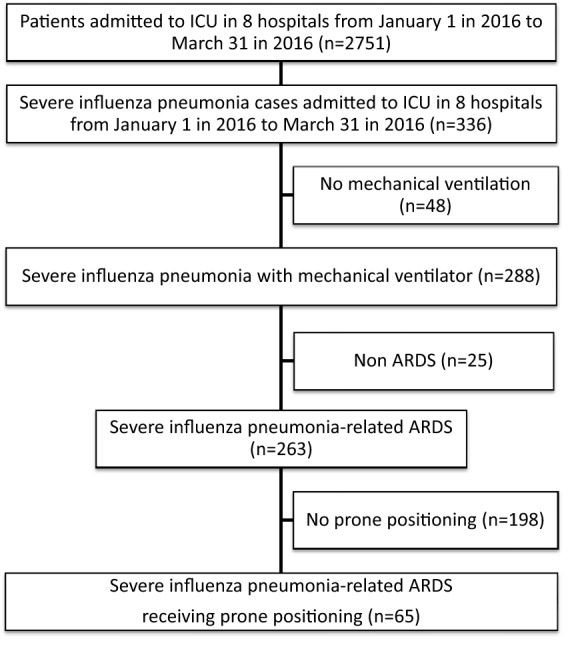
Enrollment and follow-up of the study participants. *ICU* the intensive care unit, *ARDS* acute respiratory distress syndrome

In total, 336 patients with virology-proven severe influenza pneumonia were admitted to ICUs and screened during the study period. There were 52 patients with influenza A (including H1N1 in 46 patients and H3N2 in 6 patients), 4 patients with influenza B, and 9 patients with undetermined influenza type. Of these 336 patients, 263 patients (78%) met the diagnosis of severe influenza pneumonia-related ARDS. The rates of mild, moderate and severe ARDS were 11% (28/263), 30% (79/263) and 59% (156/263), respectively. Of these 263 patients with ARDS, 65 patients (25%) receiving prone positioning were included for analysis (Fig. 1). The rate of receiving prone positioning was 18% (5/28) in mild, 15% (12/79) in moderate and 31% (48/156) in severe ARDS, respectively (*p* = 0.022).

### Characteristics of 60-day survivors and non-survivors

The characteristics of the 65 subjects according to the 60-day survivors and non-survivors are summarized in Table 1. The mean age was 57.5 ± 11.8 years, and 40 patients (62%) were male. The duration of prone positioning of survivors and non-survivors was not significantly different (3.8 ± 3.1 days vs. 3.6 ± 2.8 days, *p* = 0.729). The survivors had lower APACHE II score, PSI, creatinine level and lower rate of receiving renal replacement therapy than did non-survivors (22.4 ± 8.5 vs. 29.2 ± 7.4, *p* = 0.003; 106.6 ± 40.9 vs. 135.3 ± 48.6, *p* = 0.019; 1.2 ± 0.9 mg/dL vs. 3.1 ± 3.6 mg/dL, *p* = 0.040; and 4% vs. 42%, *p* < 0.005). Regarding the oxygenation, the mean PaO_2_/FiO_2_ ratio of these 65 patients before prone positioning was 95.9 ± 54.5 mm Hg. Before prone positioning, there were no significant differences in the PaO_2_/FiO_2_ ratio, PaCO_2_, tidal volume, PEEP, peak airway pressure, dynamic driving pressure and dynamic compliance between surviving and non-surviving patients.

**Table 1:** Characteristics of 60-day survivors and non-survivors of influenza pneumonia-related ARDS before prone positioning

Characteristics	Total patients (*n* = 65)	Survivors (*n* = 45)	Non-survivors (*n* = 20)	*p* value
Age (years)	57.5 ± 11.8	56.7 ± 13.0	59.3 ± 7.7	0.322
Gender (male/female)	40/25	27/18	13/7	0.702
BMI (kg/m^2^)	22.4 ± 4.7	27.2 ± 5.1	24.8 ± 3.5	0.057
Severity index				
APACHE II score	24.4 ± 8.7	22.0 ± 8.1	29.8 ± 7.7	0.001*
SOFA score	11.7 ± 3.6	10.9 ± 3.1	12.9 ± 3.9	0.062
PSI	115.3 ± 45.0	104.5 ± 38.9	138.4 ± 49.3	0.004*
CURB-65 score	2.2 ± 1.1	2.2 ± 1.2	2.4 ± 1.0	0.537
Influenza type				0.358
Influenza A	52 (80%)	34 (75.6%)	18 (90.0%)	
Influenza B	4 (6%)	3 (6.7%)	1 (5%)	
Undetermined	9 (14%)	8 (17.8%)	1 (5%)	
Laboratory data				
WBC (10^3^/mm^3^)	9.9 ± 6.7	9.5 ± 6.2	10.9 ± 7.8	0.433
Lactate (mg/dL)	26.8 ± 29.6	21.9 ± 23.8	36.2 ± 37.4	0.087
Albumin (g/dL)	3.1 ± 0.5	2.9 ± 0.4	2.7 ± 0.8	0.521
Creatinine (mg/dL)	1.8 ± 2.3	1.2 ± 0.9	3.1 ± 3.6	0.040*
Total bilirubin (mg/dL)	0.8 ± 0.7	0.8 ± 0.7	0.7 ± 0.9	0.694
Renal replacement therapy (*n*, %)	10 (15%)	2 (4.4%)	8 (40.0%)	0.000*
PaCO_2_ (mm Hg)	48.5 ± 17.6	47.6 ± 19.0	50.7 ± 14.2	0.510
PaO_2_/FiO_2_ ratio (mm Hg)	95.9 ± 54.5	102.3 ± 59.8	81.3 ± 37.6	0.153
Tidal volume (ml/kg PBW)	7.7 ± 2.0	7.7 ± 1.9	7.8 ± 2.4	0.985
PEEP (cm H_2_O)	13.7 ± 3.6	13.5 ± 3.9	14.2 ± 3.0	0.546
Peak airway pressure (cm H_2_O)	30.7 ± 4.2	31.0 ± 4.5	30.1 ± 3.6	0.468
Dynamic driving pressure (cm H_2_O)	17.1 ± 4.7	16.9 ± 3.5	16.6 ± 4.2	0.704
Dynamic compliance (ml/cm H_2_O)	27.1 ± 8.5	26.1 ± 7.9	29.1 ± 9.7	0.200

*ARDS* acute respiratory distress syndrome, *BMI* body mass index, *APACHE II* Acute Physical and Chronic Health Evaluation, *SOFA* Sequential Organ Function Assessment, *PSI* pneumonia severity index, *CURB*-*65* CURB-65 for pneumonia severity, *WBC* white blood cell count, *PaCO*_*2*_ atrial pressure of carbon dioxide in arterial blood, *PaO*_*2*_ atrial pressure of oxygen in arterial blood, *FiO*_*2*_ oxygen fraction in air, *PBW* predict body weight, *PEEP* positive end-expiratory pressure

All values are expressed as the number of patients (percentage) or mean ± SD

*p < 0.05: survivors versus non-survivors

### Changes in gas exchange and lung mechanics after prone positioning

The data regarding the gas exchange and lung mechanics were recorded before prone positioning and after 1-day prone positioning (Table 2). For the 30-day survivors, there were no significant differences in these parameters compared with 30-day non-survivors except for peak airway pressure. After prone positioning, the 30-day survivors had decreased peak airway pressure (− 0.5 ± 3.3 cm H_2_O) and the 30-day non-survivors had increased peak airway pressure (1.5 ± 4.1 cm H_2_O). Compared with 60-day non-survivors, the peak airway pressure and dynamic driving pressure were both decreased in 60-day survivors (− 0.6 ± 3.2 cm H_2_O vs. 1.5 ± 3.8 cm H_2_O, *p* = 0.024; − 1.5 ± 3.3 cm H_2_O vs. 0.3 ± 2.4 cm H_2_O, *p* = 0.031). The dynamic compliance was increased in 60-day survivors and decreased in 60-day non-survivors (2.0 ± 7.7 cm H_2_O vs. − 3.2 ± 8.6 cm H_2_O, *p* = 0.022).

**Table 2:** Change in gas exchange and lung mechanics between survivors and non-survivors for influenza pneumonia-related ARDS

Parameters	30-day		*p* value	60-day		*p* value
	Survivors (*n* = 48)	Non-survivors (*n* = 17)		Survivors (*n* = 45)	Non-survivors (*n* = 20)	
Δ PaO_2_ (mm Hg)	6.0 ± 53.7	6.6 ± 29.1	0.963	5.2 ± 54.2	8.4 ± 29.0	0.823
Δ PaCO_2_ (mm Hg)	− 4.7 ± 20.4	− 3.4 ± 14.3	0.817	− 5.2 ± 20.4	8.4 ± 29.0	0.823
Δ FiO_2_	− 15.9 ± 19.6	− 12.9 ± 20.5	0.597	− 17.0 ± 19.8	− 11.0 ± 19.4	0.263
Δ PaO_2_/FiO_2_ (mm Hg)	34.7 ± 83.6	30.3 ± 49.7	0.839	35.5 ± 85.9	29.2 ± 47.4	0.706
Δ P(A–a)O_2_ (mm Hg)	− 140.6 ± 173.7	− 94.3 ± 157.5	0.337	− 145.2 ± 175.4	− 90.8 ± 153.2	0.236
Δ Tidal volume (ml/kg PBW)	− 0.2 ± 1.6	− 0.5 ± 2.3	0.532	− 0.1 ± 1.6	− 0.6 ± 2.1	0.389
Δ PEEP (cm H_2_O)	1.0 ± 3.1	1.4 ± 3.5	0.672	1.1 ± 3.2	1.2 ± 3.3	0.913
Δ Peak airway pressure (cm H_2_O)	− 0.5 ± 3.3	1.5 ± 4.1	0.041*	− 0.6 ± 3.2	1.5 ± 3.8	0.024*
Δ Dynamic driving pressure (cm H_2_O)	− 1.4 ± 3.3	0.1 ± 2.4	0.106	− 1.5 ± 3.3	0.3 ± 2.4	0.031*
Δ Dynamic compliance (ml/cm H_2_O)	1.4 ± 7.9	− 2.6 ± 9.0	0.103	2.0 ± 7.7	− 3.2 ± 8.6	0.022*

*ARDS* acute respiratory distress syndrome, *Δ* change between before and after prone positioning 1 day, *PaO*_*2*_ partial pressure of oxygen in arterial blood, *PaCO*_*2*_ atrial pressure of carbon dioxide in arterial blood, *FiO*_*2*_ oxygen fraction in air, *P(A*-*a)O*_*2*_ alveolar–arterial oxygen gradient, *PEEP* positive end-expiratory pressure

All values are expressed as mean ± SD

*p < 0.05: survivors versus non-survivors

### Survival predictors in influenza pneumonia-related ARDS after prone positioning

**Fig. 2:**
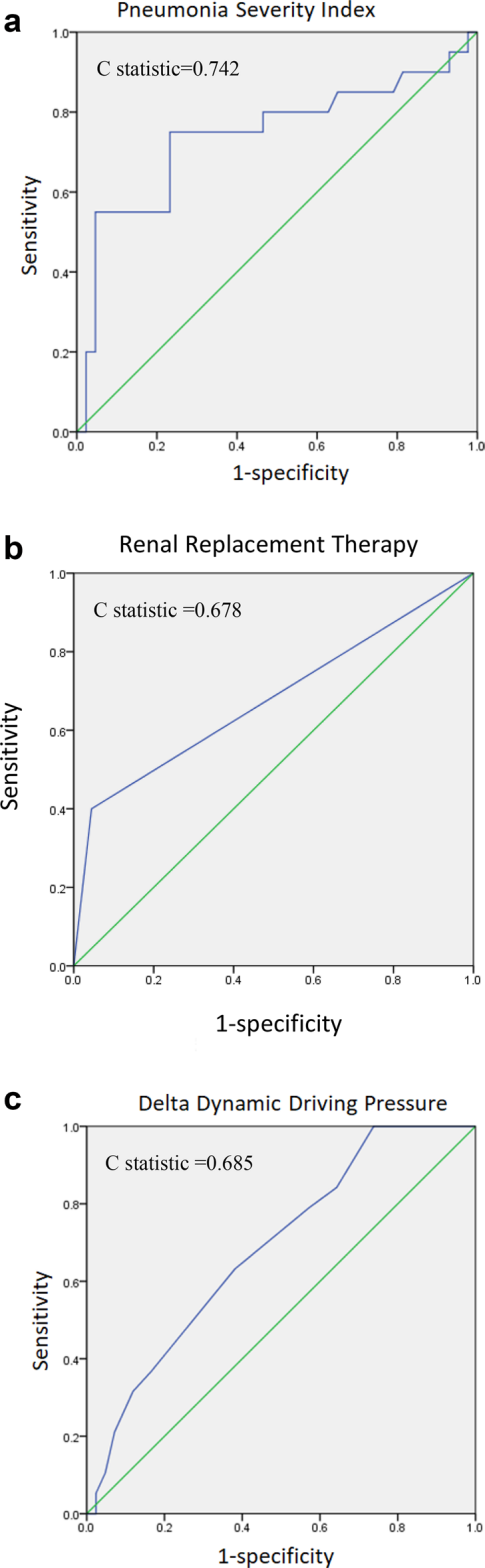
Receiver operating curves analysis and C statistics of continuous variables of predictors. **a** pneumonia severity index, **b** renal replacement therapy and **c** delta dynamic driving pressure

Univariate analysis was used to identify variables that have prognostic value for 60-day mortality, and multivariate Cox regression analysis was used to identify variables that did have significant predictive value (Table 3). Pneumonia severity index (hazard ratio 1.020, 95% confidence interval 1.009–1.032; *p* < 0.001), renal replacement therapy (hazard ratio 6.248, 95% confidence interval 2.245–17.389; *p* < 0.001) and increased dynamic driving pressure (hazard ratio 1.372, 95% confidence interval 1.095–1.718; *p* = 0.006) were identified as significant and independent predictors associated with 60-day mortality. As the collinearity between Δ dynamic driving pressure, Δ peak airway pressure and Δ dynamic compliance was statistically significant, a Cox model was constructed for each of these variables. After multiple adjustments of coupled variables, three additional Cox models were performed (Additional file 1). When Δ dynamic driving pressure and Δ peak airway pressure were analyzed two-by-two, Δ dynamic driving pressure remained significant but Δ peak airway pressure did not (model 1 in Additional file 1). When Δ dynamic driving pressure and Δ dynamic compliance were analyzed two-by-two, Δ dynamic driving pressure remained significant but Δ dynamic compliance did not (model 2 in Additional file 1). When Δ peak airway pressure and Δ dynamic compliance were analyzed two-by-two, both did not reveal significant (model 3 in Additional file 1). Receiver operating curves analysis and C statistic of variables of predictors revealed 0.742 in PSI (95% confidence interval, 0.592–0.892, *p* = 0.002), 0.678 in renal replacement therapy (95% confidence interval, 0.523–0.833, *p* = 0.023) and 0.685 (95% confidence interval, 0.547–0.823, *p* = 0.022) in delta dynamic driving pressure (Fig. 2).

**Table 3:** Cox regression analysis of clinical variables associated with 60-day mortality in influenza pneumonia-related ARDS with prone positioning

Clinical variables	Univariate		Multivariate	
	Hazard ratio (95% CI)	*p* value	Hazard ratio (95% CI)	*p* value
APACHE II score	1.089 (1.035–1.147)	0.001*	1.042 (0.982–1.106)	0.178
PSI	1.015 (1.005–1.026)	0.003*	1.020 (1.009–1.032)	< 0.001*
Renal replacement therapy	5.355 (2.159–13.281)	0.000*	6.248 (2.245–17.389)	< 0.001*
Δ Peak airway pressure (cm H_2_O)	1.143 (1.019–1.282)	0.022*	0.996 (0.822–1.208)	0.969
Δ Dynamic driving pressure (cm H_2_O)	1.147 (1.008–1.305)	0.037*	1.372 (1.095–1.718)	0.006*
Δ Dynamic compliance (ml/cm H_2_O)	0.925 (0.871–0.983)	0.011*	0.941 (0.872–1.015)	0.117

*ARDS* acute respiratory distress syndrome, *CI* confidence interval, *APACHE II* Acute Physical and Chronic Health Evaluation, *PSI* pneumonia severity index, *Δ* difference between before and after prone positioning 1 day

*p < 0.05

## Discussion

The aim of this multicenter retrospective study was to evaluate the effect of prone positioning focusing on patients with influenza pneumonia-related ARDS. After multivariate Cox regression analysis, PSI, renal replacement therapy and increased dynamic driving pressure were associated with 60-day mortality in patients with influenza pneumonia-related ARDS receiving prone positioning.

Most of the studies evaluating the effect of prone positioning were in ARDS patients with heterogeneous risk factors [14, 15]. For specific conditions such as burns, prone positioning has been demonstrated to safely implement and improve oxygenation (in burn patients with severe ARDS) in a burn intensive care unit [24]. The present study was more homogenous and specific to patients with ARDS caused by influenza pneumonia. Systematic review and meta-analysis studies in prone positioning have revealed decreased mortality in patients with severe acute hypoxemic respiratory failure, but not in less severe hypoxemia. Survival benefits were noted using a range of PaO_2_/FiO_2_ ratio thresholds up to approximately 140 mm Hg [25] or less than 200 mm Hg [26]. In the present study, the PaO_2_/FiO_2_ ratio was 95.9 ± 54.5 mm Hg before prone positioning. However, the PaO_2_/FiO_2_ ratio was not significantly different between 60-day survivors and 60-day non-survivors (102.3 ± 59.8 mm Hg vs. 81.3 ± 37.6 mm Hg, *p* = 0.153). In terms of the response of prone positioning to ARDS, the different entities of the risk factor possibly produce different outcomes. In addition to severity of hypoxemia, further clinical trials would assist in clarifying the survival benefits of prone positioning in the specific risk factors.

Some studies have shown that acute kidney injury (AKI) was common and an independent risk factor for mortality in patients with influenza A [27–30]. In patients with severe ARDS caused by H1N1 influenza pneumonia, a recent study also revealed AKI was common and demonstrated significantly increased mortality [31]. The 53% mortality rate among the 38 patients requiring renal replacement therapy was significantly higher than the 0% mortality rate among the 19 patients not requiring renal replacement therapy. The present study in patients receiving prone positioning caused by influenza pneumonia-related ARDS demonstrated that the requirement for renal replacement therapy had nearly 6 times the mortality rate (hazard ratio 6.248) than patients not requiring renal replacement therapy. In order to reduce the mortality in patients with severe ARDS caused by H1N1 influenza pneumonia, it is important to prevent development of AKI and need for renal replacement therapy by avoiding nephrotoxic agents and supplying sufficient renal perfusion and oxygenation.

Amato and colleagues analyzed 9 randomized controlled trials in ARDS patients and demonstrated that driving pressure was the strongest predictor of mortality [32]. A secondary analysis of data from 787 ARDS patients enrolled in two independent randomized controlled trials revealed that when ventilating patients with low tidal volume, driving pressure was a risk factor for death in ARDS patients, as was plateau pressure or compliance of respiratory system [33]. Airway driving pressure was significantly related to lung stress and could detect lung over-stress with acceptable accuracy (*r*^2^ = 0.581 *p* < 0.0001 and *r*^2^ = 0.353 *p* < 0.0001 at 5 and 15 cm H_2_O of PEEP) in ARDS patients [23]. Furthermore, the APRONET study on prone positioning of ARDS patients found that prone positioning was associated with low complication rates, significant increase in oxygenation, and a significant decrease in driving pressure [14 (11–17 cm H_2_O) to 13 [10–16] cm H_2_O, *p* = 0.04] [34]. Our previous study for severe ARDS patients with ECMO revealed that higher dynamic driving pressure [hazard ratio 1.070 (1.026–1.116), *p* = 0.002] during the first 3 days of ECMO was one of the factors independently associated with ICU mortality [35]. The present study in influenza pneumonia-related ARDS patients receiving prone positioning also found that increased dynamic driving pressure (hazard ratio 1.372, 95% confidence interval 1.095–1.718; *p* = 0.006) was identified as one of the independent predictors associated with 60-day mortality. It was suggested that ventilatory support with lung-protective strategy with low tidal volume and optimal PEEP level be applied, and these be then adjusted according to the driving pressure, ideally less than 15 cm H_2_O, although this limit should be addressed in future studies [36]. Despite some studies associating driving pressure with physiological and clinical outcomes, it is necessary to evaluate the driving pressure as a primary end point during ventilatory setting in ARDS patients in the near future.

The LUNG SAFE study showed that the use of prone positioning actually depended on the severity of hypoxemia, from 1% in mild to 5.5% in moderate and to 16.3% in severe ARDS [37]. A prospective international prevalence study (the APRONET study, ARDS Prone Position Network) found that the rates of prone positioning were up to 5.9%, 10.3% and 32.9% in mild, moderate and severe ARDS [30]. In our study, the rates of prone positioning were 18%, 15% and 31% in mild, moderate and severe ARDS, respectively. The substantially different rates in the use of the prone positioning may reflect the management bias of prone positioning in patients with ARDS between the different studies. Furthermore, among our eight involved hospitals, the rate of prone positioning varied from 0% (0/37) to 67% (2/3) and the bias even existed between different hospitals in the same study. It is important to be homogenous on the indication and management in the selected prone position as one of the standard interventions in severe ARDS.

This study has some limitations. Firstly, since this study is retrospective, some patients or data might be missing. Secondly, the primary end point of this study was 60-day mortality, and the value of computed power was 0.585. This was a retrospective study, and 65 patients with severe ARDS receiving prone positioning were analyzed. Although more patients were needed to increase the power of this study, the limitation was from the nature of retrospective study within a 3-month period. Thirdly, prone positioning is not a routine intervention in the management of ARDS and has no standard procedure such as how many hours a day, how to perform it or how to protect the patients. In this study, even though every patient had prone positioning for more than 16 h a day, the exact duration showed little difference between each hospital. Fourthly, the change in physiological measurements pertains to a difference between supine and prone position, and hence, the impact of chest wall is not taken into account. Finally, in this study, we focused on influenza-related ARDS patients, and whether the result can be extrapolated to all patients with ARDS is unknown, requiring further investigation. To confirm the benefit of prone positioning in ARDS especially in influenza pneumonia, further prospective randomized control studies are needed with strict standard procedures and patient selection.

## Conclusions

This study was designed to evaluate the effect of prone positioning in influenza pneumonia-related ARDS patients. After multivariate Cox regression analysis, it was found that PSI, renal replacement therapy and increased dynamic driving pressure were associated with 60-day mortality in patients with influenza pneumonia-related ARDS receiving prone positioning.

## Additional file

**Additional file 1.** Cox regression analysis of clinical variables associated with 60-day mortality ininfluenza pneumonia-related ARDS with prone positioning.

## Abbreviations

ARDS: acute respiratory distress syndrome
APACHE: Acute Physiology and Chronic Health Evaluation
PSI: pneumonia severity index
ICU: intensive care units
TSIRC: Taiwan Severe Influenza Research Consortium
RT-PCR: reverse transcriptase polymerase chain reaction
PEEP: positive end-expiratory pressure
SOFA: Sequential Organ Failure Assessment
AKI: acute kidney injury
CURB-65: Confusion, Urea, Respiratory rate, Blood pressure and age
WBC: white blood cell count
CRP: C-reactive protein
P(A–a)O_2_: alveolar–arterial oxygen gradient
PBW: predict body weight
BMI: body mass index
CI: confidence interval
