# Supplementary material for: Predictors of survival in patients with influenza pneumonia-related severe acute respiratory distress syndrome treated with prone positioning
Source: Ann Intensive Care. 2018 Sep 24;8:94. doi: 10.1186/s13613-018-0440-4 (PMC6153196; doi:10.1186/s13613-018-0440-4)
Supplement: Supplementary file 1 — Additional file 1. Cox regression analysis of clinical variables associated with 60-day mortality ininfluenza pneumonia-related ARDS with prone positioning. [file 13613_2018_440_MOESM1_ESM.pdf]

Supplementary Material 1: Cox regression analysis of clinical variables associated with 60-day mortality in influenza pneumonia-related ARDS with prone positioning

Model 1: Couple is Δ Peak airway pressure and Δ Dynamic driving pressure

**Clinical variables**	**Univariate**		**Multivariate**	
	**Hazard ratio (95% CI)**	**p value**	**Hazard ratio (95% CI)**	**p value**
APACHE II score	1.089 (1.035-1.147)	0.001*	1.042 (0.982-1.106)	0.178
PSI	1.015 (1.005-1.026)	0.003*	1.020 (1.009-1.032)	<0.001*
Renal replacement therapy	5.355 (2.159-13.281)	0.000*	6.248 (2.245-17.389)	<0.001*
Δ Peak airway pressure (cm H_2O)	1.143 (1.019-1.282)	0.022*	1.064 (0.915-1.238)	0.422
Δ Dynamic driving pressure (cm H_2O)	0.925 (0.871-0.983)	0.011*	1.369 (1.090-1.719)	0.007*

ARDS: acute respiratory distress syndrome; *CI*: confidence interval; *APACHE II*: Acute Physical and Chronic Health Evaluation; *PSI*: pneumonia severity index; *Δ*: difference between before and after prone positioning 1 day.

*p< 0.05

Supplementary Material 1: Cox regression analysis of clinical variables associated with 60-day mortality in influenza pneumonia-related ARDS with prone positioning

Model 2: Couple is Δ Dynamic driving pressure and Δ Dynamic compliance

**Clinical variables**	**Univariate**		**Multivariate**	
	**Hazard ratio (95% CI)**	**p value**	**Hazard ratio (95% CI)**	**p value**
APACHE II score	1.089 (1.035-1.147)	0.001*	1.042 (0.982-1.106)	0.178
PSI	1.015 (1.005-1.026)	0.003*	1.020 (1.009-1.032)	<0.001*
Renal replacement therapy	5.355 (2.159-13.281)	0.000*	6.248 (2.245-17.389)	<0.001*
Δ Dynamic driving pressure (cm H_2O)	1.147 (1.008-1.305)	0.037*	1.372 (1.095-1.718)	0.006*
Δ Dynamic compliance (ml/cm H_2O)	0.925 (0.871-0.983)	0.011*	0.941 (0.872-1.015)	0.117

ARDS: acute respiratory distress syndrome; *CI*: confidence interval; *APACHE II*: Acute Physical and Chronic Health Evaluation; *PSI*: pneumonia severity index; *Δ*: difference between before and after prone positioning 1 day.

*p< 0.05

Supplementary Material 1: Cox regression analysis of clinical variables associated with 60-day mortality in influenza pneumonia-related ARDS with prone positioning

Model 3: Couple is Δ Peak airway pressure and Δ Dynamic compliance

Clinical variables	Univariate		Multivariate	
	Hazard ratio (95% CI)	p value	Hazard ratio (95% CI)	p value
APACHE II score	1.089 (1.035-1.147)	0.001*	1.042 (0.982-1.106)	0.178
PSI	1.015 (1.005-1.026)	0.003*	1.020 (1.009-1.032)	<0.001*
Renal replacement therapy	5.355 (2.159-13.281)	0.000*	6.248 (2.245-17.389)	<0.001*
Δ Peak airway pressure (cm H_2O)	1.143 (1.019-1.282)	0.022*	0.987 (0.820-1.188)	0.899
Δ Dynamic compliance (ml/cm H_2O)	0.925 (0.871-0.983)	0.011*	0.908 (0.851-1.012)	0.076

ARDS: acute respiratory distress syndrome; *CI*: confidence interval; *APACHE II*: Acute Physical and Chronic Health Evaluation; *PSI*: pneumonia severity index; *Δ*: difference between before and after prone positioning 1 day.

*p< 0.05
